# Actinobacteria–Plant Interactions in Alleviating Abiotic Stress

**DOI:** 10.3390/plants11212976

**Published:** 2022-11-04

**Authors:** Manik Prabhu Narsing Rao, Karan Lohmaneeratana, Chakrit Bunyoo, Arinthip Thamchaipenet

**Affiliations:** 1 Department of Genetics, Faculty of Science, Kasetsart University, Bangkok 10900, Thailand; 2 Omics Center for Agriculture, Bioresources, Food and Health, Kasetsart University (OmiKU), Bangkok 10900, Thailand; 3 Interdisciplinary Graduate Program in Bioscience, Faculty of Science, Kasetsart University, Bangkok 10900, Thailand

**Keywords:** plants, actinobacteria, plant growth-promoting bacteria, environmental stressors, multi-omics

## Abstract

Abiotic stressors, such as drought, flooding, extreme temperature, soil salinity, and metal toxicity, are the most important factors limiting crop productivity. Plants use their innate biological systems to overcome these abiotic stresses caused by environmental and edaphic conditions. Microorganisms that live in and around plant systems have incredible metabolic abilities in mitigating abiotic stress. Recent advances in multi-omics methods, such as metagenomics, genomics, transcriptomics, and proteomics, have helped to understand how plants interact with microbes and their environment. These methods aid in the construction of various metabolic models of microbes and plants, resulting in a better knowledge of all metabolic exchanges engaged during interactions. Actinobacteria are ubiquitous and are excellent candidates for plant growth promotion because of their prevalence in soil, the rhizosphere, their capacity to colonize plant roots and surfaces, and their ability to produce various secondary metabolites. Mechanisms by which actinobacteria overcome abiotic stress include the production of osmolytes, plant hormones, and enzymes, maintaining osmotic balance, and enhancing nutrient availability. With these characteristics, actinobacteria members are the most promising candidates as microbial inoculants. This review focuses on actinobacterial diversity in various plant regions as well as the impact of abiotic stress on plant-associated actinobacterial diversity and actinobacteria-mediated stress mitigation processes. The study discusses the role of multi-omics techniques in expanding plant–actinobacteria interactions, which aid plants in overcoming abiotic stresses and aims to encourage further investigations into what may be considered a relatively unexplored area of research.

## 1. Introduction

Abiotic stress is any environmental factor limiting plant growth and productivity [1]. It is brought on by environmental factors such as water, salt, light, temperature, and nutrients, which can significantly inhibit plant growth, yield, and survival [2]. It was estimated that environmental factors could reduce crop production by up to 70% [3]. Abiotic stress includes drought, flooding, temperature fluctuations, high soil salinity, and metal toxicity [4]. Plant responses to abiotic stress are both reversible and irreversible [5].

Drought stress is one of the most significant abiotic stresses that affect plant growth and development. Plants are drought-stressed when available water in the soil is reduced to critical levels and contributes to continuous water loss [6]. Reduction of leaf water potential, turgor pressure, stomatal closure, and cell development are all signs of drought stress in plants [7]. Numerous physiological and biochemical processes, including photosynthesis, chlorophyll synthesis, nutrient metabolism, ion uptake and translocation, respiration, and carbohydrate metabolism, are also reduced by drought stress [8]. In contrast to drought, excess water is another problem for plant growth and development [9]. Water stress causes a decrease in leaf water potential and stomatal opening, which leads to the downregulation of photosynthesis-related genes and decreased CO_2_ availability [7,10]. Furthermore, temperature (high and low) strongly influences the metabolic activity of plants [11]. Cold-driven rigidification and heat-driven fluidization can cause membrane dysfunction, as exemplified by protein deactivation and ion leakage [12,13].

Soil salinity is another severe issue for plants, reducing crop yield worldwide [14]. Salt stress causes cellular dehydration, leading to osmotic stress and water removal from the cytoplasm, decreasing cytosolic and vacuolar volumes [6]. Osmotic stress causes various physiological changes, including membrane disruption, nutrient imbalance, impaired ability to detoxify reactive oxygen species, differences in antioxidant enzymes, decreasing photosynthetic activity and stomatal aperture, and accumulation of Na^+^ and Cl^−^ ions in the tissues of plants [15]. Due to industrial waste and sewage disposal, heavy metals have long been accumulating in soils [16]. Plants exposed to heavy metals experience altered membrane permeability, enzyme inhibition, photosystem inactivation, and disturbances in mineral metabolism [17,18].

Plants have evolved various mechanisms to deal with abiotic stresses, one of which is the use of microbes, which is an effective, environmentally friendly, and economically viable method [19]. Microorganisms represent a natural soil microflora with high metabolic capacities for growth promotion and resistance to abiotic stresses [20]. Microbes may, directly and indirectly, contribute to plant growth and stress resistance by various mechanisms, including increased nutrient availability, prevention of diseases, nitrogen fixation, and production of hydrolytic enzymes and phytohormones [21,22,23,24]. Plants release numerous signals/clues that allow effective communication between plants and microorganisms [25]. Plants actively recruit their microorganisms from surrounding microbial reservoirs such as soil, rhizosphere, and phyllosphere [26]. The enrichment of microorganisms by the plant is not random, but rather a targeted process [22]. Several factors (such as geographic regions, soil abiotic factors, and climate conditions) may explain the dramatic variation in the correlation between microbial and plant diversity [27,28,29,30]. Even within the plant, different plant organs and plant stages are dominated by different microbes [31]. Some dominant bacterial phyla associated with plants are Acidobacteria, Verrucomicrobia, Bacteroidetes, Proteobacteria, Planctomycetes, and Actinobacteria [26,32].

Actinobacteria are Gram-positive bacteria common in soil conditions and constitute one of the largest bacterial phyla [33]. Actinobacteria exhibit a variety of characteristics that are similar to fungi [34]. The first hierarchal phylogenetic clustering of members of the Actinobacteria was provided by Stackebrandt et al. [35]. Actinobacterial taxonomy has evolved throughout time, with the most recent roadmap dividing the phylum into six classes, 46 orders, and 79 families, with 16 new orders and 10 new families [36]. Actinobacteria can form complex structures such as spores, spore chains, sporangia, and sporangiospores [33]. The growth of substrate mycelium, the position of the spore, the quantity of spores, the surface structures of the spore, the form of the sporangia, and whether or not the sporangiospore has flagella are all key morphological aspects of actinobacteria classification [37]. Actinobacteria have a wide range of morphologies, including rod shape (*Acidiferrimicrobium*) [38], coccoid (*Micrococcus*) [39], rod-coccoid (*Arthrobacter*) [40], and bent rods (*Sinomonas*) [41] forms, as well as fragmenting hyphal forms (*Nocardia*) and forms with permanently differentiated branched mycelia (e.g., *Streptomyces* and *Frankia*). Some develop elongated filaments on the substrate but no true mycelium (*Rhodococci*) [33,42], whereas some do not produce mycelia at all (*Corynebacterium*) [43], while some distinguished by the production of branched substrate hyphae that break up into flagellated motile elements (*Oerskovia*) [33,44]. Many actinobacterial members can invade plant roots and surfaces [45]. Furthermore, they can produce extracellular compounds that allow them to outcompete phytopathogens and act as plant growth regulators.

The wide distribution of actinobacterial genera among plant growth-promoting bacteria, their high abundance in soil, having genomes dedicated to secondary metabolite production, and the number of plant growth promoter genera reported from actinobacteria, make them ideal candidates for development as microbial inoculants [46,47]. Although many earlier reviews focused [48,49] on plant growth promotion (PGP) traits of microorganisms and abiotic stress mitigation abilities, this review focuses on actinobacterial diversity in various plant regions and the impact of abiotic stress on plant-associated actinobacterial diversity and actinobacteria-mediated stress mitigation processes. The role of multi-omics approaches in expanding our knowledge of plant–actinobacteria interactions that aid plants in overcoming abiotic stresses are also discussed.

## 2. Actinobacteria Diversity Associated with Plants and Plant Growth Promotion

The rhizosphere is the soil zone surrounding plant roots that influence the biological and chemical properties of the soil [50]. Bacterial concentrations in the rhizosphere are about 10–1000 times greater than in bulk soil [51]. The rhizosphere is in direct contact with the plant roots and is actively nourished by a complex mixture of carbon/nutrient sources given by the plant, such as amino acids, sugars, and other nutrients [52].

Actinobacteria are dominant in the rhizosphere, and their contributions to soil systems have a significant economic influence [53]. They are considered rhizosphere competent because they can use plant nutrients found in the rhizosphere and stimulate plant development following inoculation [54]. Various rhizospheric actinobacterial members, with their PGP, nutrient cycling, anti-phytopathogenic activity, and ability to thrive in harsh conditions, have been reported for a wide range of plants. Among various actinobacterial members, *Streptomyces* are commonly found in soil and can colonize the rhizosphere and root tissues with PGP activity [55]. For example, *Streptomyces* sp. isolated from wheat rhizosphere showed PGP activities, namely, phosphate solubilization, production of indole-3-acetic acid (IAA), siderophore, phytase, and chitinase, as well as utilization of sugars in the rhizosphere [54]. *Streptomyces lydicus* WYEC108, that colonized and sporulated within the nodule’s surface cell layers of pea root, influenced nodulation by increasing the average size of the nodules, improving the vigor of bacteroids within the nodules by enhancing nodular iron and possibly other soil nutrient assimilation [56]. The impact of *Streptomyces* spp. isolated from the rhizosphere on five legumes (soybean, kidney bean, chickpea, lentil, and pea) demonstrated that soil microbial populations were boosted, while soil nutrients and organic matter content were also increased [57]. Soil enrichment with *Streptomyces* sp. boosted photosynthesis, which subsequently increased legume production. *Streptomyces* sp. also boosted nitrogen availability in soil, legume tissue, and seeds, which activated critical nitrogen metabolizing enzymes such as glutamine synthetase, glutamate synthetase, and nitrate reductase. In addition to higher amounts of nitrogen-containing amino acids in actinobacterial-treated legume seeds, significant quantities of sugar, organic acids, and fatty acids, as well as antioxidant phenolics, minerals, and vitamins were also observed [57]. Members of the genus *Streptomyces* and a few *Kitasatospora* were predominantly isolated from the yam rhizosphere and promoted the growth of *Arabidopsis* seedlings [58]. All of them produced IAA and siderophores, half exhibited tricalcium phosphate-solubilizing activity, and 20% harbored 1-aminocyclopropane-1-carboxylic acid (ACC) deaminase activity. Not only *Streptomyces* spp., but other actinobacterial members have also been reported from the rhizosphere as having PGP activity. For example, a multiple growth-promoting *Tsukamurella tyrosinosolvens* (isolated from the rhizosphere soil of tea plants) was reported to secrete various organic acids, such as lactic acid, maleic acid, and oxalic acid; solubilize phosphate and produce IAA and siderophore to enhance plant growth [59].

**Figure 1:**
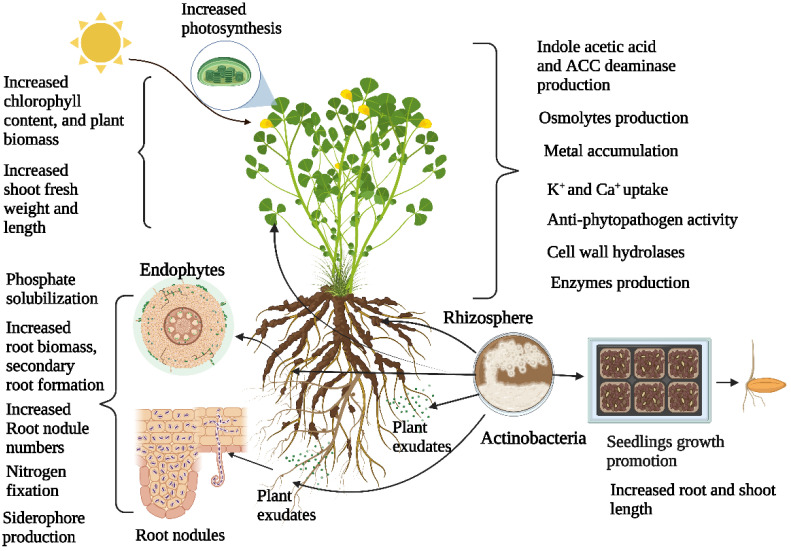
Role of Actinobacteria in various plant parts.

The actinobacterial strains also showed PGP activity in field trials. *Streptomyces corchorusii* UCR3-16 isolated from rice rhizosphere was tested for PGP activity in field trials utilizing a talcum-based powder formulation [60]. *S. corchorusii* UCR3-16 significantly improved shoot length, shoot and root weight, total grain yield, and grain weight in rice. The sheath blight disease in rice leaves was also dramatically decreased by the talcum formulation [60]. Similarly, *Streptomyces* sp. CAI-8 isolated from rhizosphere soils of chickpeas under field conditions showed an increment in nodule numbers, root weight, stover yield, and grain yield [61]. Antifungal activity of *Streptomyces* spp. VV/R1 and VV/R4 isolated from the rhizosphere were tested for PGP in a field trial [62]. Both strains significantly reduced the infection rates of several fungal pathogens (*Dactylonectria* sp., *Ilyonectria* sp., *Phaeoacremonium chlamydospora*, and *Phaeoacremonium minimum*) that caused young grapevine. These isolates also significantly reduced the mortality level of grafted plants in the nursery [62]. The overall PGP properties of actinobacterial strains isolated from the rhizosphere are shown in Figure 1.

Microbial endophytes have co-evolved along with plants by colonizing apoplast and symplast regions of the host plant [63]. Among the endophytes, actinobacterial members are excellent dwellers in plant tissues, and their ecology in plants is exceptionally diverse [64]. It has been documented that actinobacterial members can colonize any tissue or organ of the host plant and they are prevalent in the roots, somewhat plentiful in the branches, and rare in the leaves [50,65,66]. The first actinobacterial endophyte to be isolated was *Frankia,* which is a nitrogen-fixing microorganism that induces nodulation on several angiosperm plant families and has received substantial attention due to its role in the nitrogen economy of its hosts [45,67]. *Frankia* sp. DDNSF-01 and *Frankia casuarinae* DDNSF-02 isolated from the root nodules of *Casuarina* sp. showed activity against phytopathogens including *Pseudomonas* sp. and *Colletotrichum* sp. in addition to the production of IAA, siderophore, and ammonia, as well as phosphate solubilization [68].

In general, the endophytic actinobacterial members were the most commonly isolated from roots, followed by stems, and leaves [69]. *Streptomyces* spp. were the predominant species, followed by *Microbispora*, *Micromonospora*, *Nocardioides*, *Nocardia*, and *Streptosporangium* which were commonly found among the culturable endophytic actinobacteria [70,71]. In the past few years, various endophytic actinobacterial members were reported for PGP activity. For example, endophytic *Streptomyces* and *Amycolatopsis* isolated from *Camellia oleifera* increased the growth of *C. oleifera* seedlings [72]. Similarly, *Streptomyces* spp. and *Amycolatopsis* spp. were used in the hydroponic germination of wheat seeds, and promoted plant growth in terms of root and stem parts [72]. The genus *Streptomyces* was mostly dominant among the isolates recovered from leaf, stem, and root samples of tea, including *Actinomadura*, *Kribbella*, *Nocardia*, *Kytococcus*, *Leifsonia*, *Microbacterium*, *Micromonospora*, *Mobilicoccus*, *Mycobacterium*, *Nocardiopsis*, *Piscicoccus*, and *Pseudonocardia*, whereas *Mobilicoccus* and *Piscicoccus* were reported for the first time as plant endophytes [73]. These strains produced IAA and ACC deaminase, exhibited antimicrobial activity, and carried polyketide synthase (PKS-I and PKS-II) and non-ribosomal peptide synthetase genes [73].

Endophytic actinobacterial members also showed enhanced growth when co-inoculated with other microbial strains. Co-inoculation of endophytic *Microbispora* sp. CP56, *Actinomadura* sp. CP84B, *Streptomyces* spp. CP200B, and CP21A with *Mesorhizobium cicero* in chickpea seedlings showed growth promotion and enhancement of the rhizobia–chickpea symbiosis by increasing nodulation-related biological processes such as rhizobial chemotaxis, biofilm formation, and *nod* gene expression [74]. When PGP endophytic strains *Microbispora* sp. GKU 823 and *Streptomyces* sp. GKU 895 were co-inoculated with the PGP diazotrophs *Bacillus* sp. EN-24 and *Enterobacter* sp. EN-21, the growth of sugarcane was increased when compared with individual inoculation [75]. In addition, endophytic *Streptomyces* spp. isolated from plant roots grown in contaminated soil showed PGP features such as phosphate solubilization and production of ACC deaminase, IAA, biosurfactant, and siderophores with the ability of phytoremediation by degradation of petroleum increasing up to 98% after 7 days of incubation [76].

## 3. Effect of Abiotic Stress on Actinobacterial Diversity in Plant Microbiome

**Figure 2:**
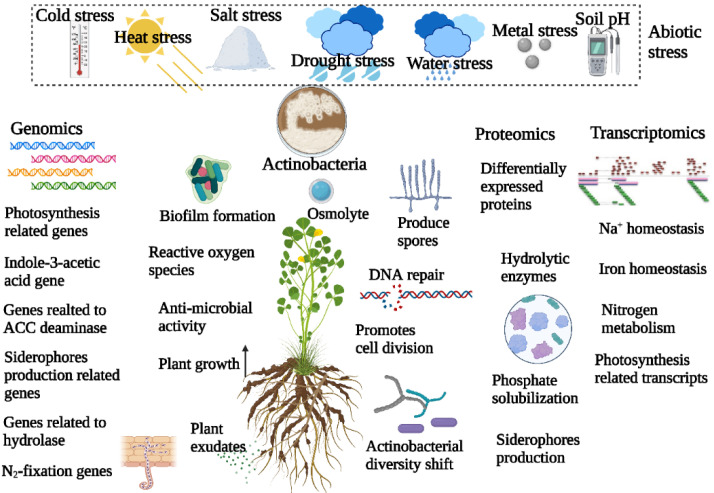
Actinobacteria-mediated abiotic stress mitigation in plants. The effect of abiotic stress on actinobacteria–plant interaction was evaluated by various omics approaches.

Interactions between microbes and plants are essential for the survival and adaptation of both partners in the abiotic environment [77]. Several studies have been conducted to understand the impact of abiotic stresses on actinobacterial diversity and their functions associated with plants (Figure 2).

Drought stress is a serious and growing issue in agriculture, affecting plant growth and development [78]. The effect of drought stress (three-week-long drought treatments) on rice root-associated microbiomes have been evaluated [79]. Actinobacteria have been reported to be the most highly represented phylum in both the rhizosphere and endosphere communities under drought stress, with multiple families of the order *Actinomycetales* being identified across all soil types [78]. Various Actinobacteria from the classes *Thermoleophilia* and *Acidimicrobiia* were abundant in the rhizosphere [78]. Such abundance of those Actinobacteria associated with plants could be explained by a number of mechanisms, including their growth habit, ability to produce stress-resistant spores, osmoprotectant production, biofilm formation, and upregulation of DNA repair [79,80].

A series of field and greenhouse experiments have been carried out to obtain spatially resolved measurements of the compositional shifts within the millet root microbiome that occurs in response to drought. According to the findings, the degree of the drought was related to the levels of actinobacterial enrichment in four millet species [81]. Drought-induced actinobacterial enrichment occurred along the length of the root, while the response was localized to drought-affected areas of the root. It was discovered that Actinobacteria were depleted in dead root tissue, implying that saprophytic activity was not the primary cause of the observed shifts in drought-treated roots [81]. The effect of drought stress on the bacterial community has been dynamically studied in grass root microbiomes (wheat, rye, barley, oat, *Brachypodium*, tall fescue, sorghum, Indian grass, *Miscanthus*, plume grass, maize, millet, and tef) [82]. Under drought stress, Actinobacteria abundance was found to be increased across all host species with the greatest abundance in the roots, in which a 3.1-fold increase was noticed compared to 2.3 and 1.5-fold increases in the rhizospheres and soils, respectively [82]. The relative increase in abundance has been hypothesized to be due to DNA replication and cell division inherent to sporulation or other bacterial lineages that indeed perish under these conditions. The results of genome analysis and quantification of absolute abundance of Actinobacteria in roots under drought and normal conditions were carried out using digital droplet PCR. The results suggested that neither decreases in the abundance of other taxa nor sporulation were likely to be the only factors responsible for Actinobacteria enrichment during drought stress [82]. Similarly, the study of the microbial community structure of drought-treated peanuts revealed that the relative abundance of Actinobacteria increased dramatically in drought-treated soil during the seedling and podding stages [83].

Soil salinity is severe environmental stress that can alter the composition of the rhizosphere soil bacterial community. The bacterial community’s distribution patterns in the rhizosphere of Jerusalem artichoke roots under different salinity stress conditions were evaluated [84]. In all conditions, Actinobacteria was among the four most dominant phyla and shared a similar percentage in either low or high salt concentration. The high abundance was speculated to be due to the proliferation of halophilic bacteria in soil [84]. Evaluation of the impact of salt stress on groundnut growth and rhizosphere microbial community structure demonstrated that the Actinobacteria number decreased after salt treatment [85]. The impact of saline stress on soil bacterial communities and Cd availability was studied in long-term wastewater-irrigated field soil [86]. An increment in soil salinity increased Cd availability. Actinobacteria were dominantly found in saline stress-treated soils, particularly members in the family *Nitriliruptoraceae* which was proposed as the most sensitive biomarker responsive to high salinity [86]. The abundance of *Nitriliruptor* species was speculated to be due to its haloalkaliphilic ability and presumably to better adaptation to high alkali environments [86].

Soil pH is another critical factor that has a significant impact on soil biology, chemistry, and physical processes, all of which have direct effects on plant growth and development [87]. The concentration of hydrogen ions, which defines soil pH, controls the entire chemistry of plant nutrition colloidal solutions. Multiple stressors, including hydrogen ion toxicity, nutritional imbalance, toxicities, and deficiencies, are caused in plants above certain pH levels [87]. The Park Grass experiment has been conducted to investigate the reaction of biological communities to long-term treatments and the related changes in soil characteristics, particularly soil pH [88]. Soil pH was positively correlated with the most abundant actinobacterial genera, namely *Mycobacterium*, *Nocardioides*, *Streptomyces*, *Micromonospora*, *Solirubrobacter*, and *Methylibium* [88]. Similarly, Actinobacteria abundance decreased with lower pH and increased at higher pH [89]. *Streptomyces* strains CAI-24, CAI-121, CAI-127, KAI-32, and KAI-90 were tested for plant growth promotion under greenhouse and field conditions of sorghum and rice. These strains were reported to express PGP activity at pH values between 5 and 13 [90].

Cold stress severely curbs the physiological and biochemical reactions in the plant cell [91]. The effect of cold stress on plant-associated microbes has also been studied. The endophytic bacterial communities of two mulberry cultivars (X792 resistant to low temperature and DS sensitive to low temperature) were studied under cold conditions in January and February [92]. Proteobacteria and Actinobacteria were identified as the two phyla that predominated in all samples. Proteobacteria predominated throughout all other samples, while Actinobacteria was the major phylum in the DS stem in both January and February samples. Except for the stem in February, all cultivar X792 samples were reported to have larger relative abundances of Proteobacteria than the DS samples, but Actinobacteria exhibited the opposite pattern. No significant difference in the relative abundance of Actinobacteria between mulberry cultivars was noticed, while there was a significant increase in the relative abundance of Actinobacteria in the stem of January compared with February. Proteobacteria was reported to increase significantly in stems compared to roots in February, while Actinobacteria decreased*. Rhodococcus* was reported as the predominant genus in the stem sample from the January sample of DS and X792. Moreover, the relative abundance of *Rhodococcus* was significantly higher, in the stem sample from the January sample of X792 compared with that in February of X792. The results also indicated a greater influence of the temperature on the endophytic bacterial content of the stem compared with that of the root [92].

Heavy metal contamination of agricultural soil has become a critical environmental concern due to its negative ecological effects, widespread occurrence, and acute and chronic toxic effects on plants [93]. Heavy metal contamination also affects the microbial diversity associated with plants. The effect of mixed heavy metal (Cd, Pb, and Zn) stress on the bacterial diversity and community composition of paddy field soils has been evaluated [94]. Under metal stress, the top two abundant phyla were Proteobacteria and Actinobacteria, but the bulk and rhizosphere soils at the heavily polluted site had a higher relative abundance of Proteobacteria, whereas the unpolluted site had a higher diversity of Actinobacteria [94]. Bacterial diversity was examined from the heavy metal-contaminated rhizosphere of the metal-hyperaccumulating plant (*Thlaspi caerulescens*) to that of contaminated bulk soil by comparing 16S rDNA and reverse-transcribed 16S rRNA libraries [95]. The dominant groups were Actinobacteria, Proteobacteria, Acidobacteria, and Planctomycetales. The study demonstrated that, except for Actinobacteria, bacterial taxa that were dominant in the rDNA library were less dominant in the rRNA library, indicating that only a portion of the bacterial community was presumably metabolically active in the heavy metal-contaminated soil. The Actinobacteria was dominated by the genus *Rubrobacteria*, implying that members of this group may be metabolically active in heavy metal-polluted soils [95].

Plants also experience multiple abiotic stresses; in this regard, the effects of heat and drought stresses on the root microbiome of *Sorghum bicolor* have been studied [96]. The relative abundances of members of the phylum Actinobacteria increased, which was particularly correlated with drought and increased temperature in both plant roots and the surrounding soil mixture. At the genus level, *Streptomyces* spp. largely dominated the root fraction, especially when high temperature was combined with drought [96]. The above studies suggest that abiotic stress greatly influences the actinobacterial diversity associated with plants.

## 4. Role of Actinobacteria in Overcoming Plant Abiotic Stress

Salinity, water shortage, and heavy metal contamination of soil and water are major problems for plant growth, crop quality, and yield, and result from a variety of physiological and metabolic changes in plants, including nutritional imbalance, water uptake inhibition, seed germination, photosynthesis, and a reduction in growth [95,97,98]. Ethylene is one of the major plant hormones that mediate the response to abiotic stresses [99]. Abiotic stress causes ethylene synthesis through the actions of ACC synthase (ACS) and ACC oxidase (ACO) in the pathway which consequently controls downstream stress-responsive genes [100]. Plant ethylene can be, however, reduced by ACC deaminase-producing plant-associated bacteria that convert ACC to ammonia and *α*-ketobutyrate, while the level of stress ethylene was consequently reduced [101]. Below are examples of PGP actinobacteria enhancing plant growth and tolerance of environmental stresses by the production of plant hormones, antioxidants, compatible solutes, and ACC deaminase.

Halotolerant actinobacteria such as *Micrococcus yunnanensis*, *Corynebacterium variabile*, and *Arthrobacter nicotianae* exhibited ACC deaminase activity that significantly promoted the growth of Canola plants under salt stress [102]. *Streptomyces* sp. PGPA39 alleviated salt stress in tomato plants by increasing chlorophyll content and plant biomass and reducing leaf proline content [103]. *Streptomyces* sp. KLBMP S0051 and *Micromonospora* sp. KLBMP S0019 isolated from coastal salt marsh rhizosphere, promoted seed germination, while *Micromonospora* sp. KLBMP S0019 significantly enhanced seedling growth under NaCl stress [104]. Endophytic *Streptomyces* sp. GMKU 336 significantly increased growth, chlorophyll, proline, K^+^, and water content; but decreased ethylene, reactive oxygen species (ROS), and Na^+^ in rice under salt stress by the expression of *acdS* encoding ACC deaminase [23]. The changes in responsive physiology were correlated to the high expression of genes involved in osmotic balance, Na^+^ transporters, calmodulin, and antioxidant enzymes; and the downregulation of genes involved in the ethylene pathway in salt stress inoculated rice. Moreover, overexpression of *acdS* in *Streptomyces venezuelae* significantly boosted the salt tolerance of rice by increasing proline and reducing ethylene and Na^+^ content compared with that of the original strain [105]

PGP *Streptomyces coelicolor* DE07, *Streptomyces olivaceus* DE10, and *Streptomyces geysiriensis* DE27 were reported to promote the growth of wheat under water stress conditions [21]. When drought-tolerant *Streptomyces pseudovenezuelae* and *Arthrobacter arilaitensis* were used as bio-inoculants, they increased growth and reduced the negative effects of drought stress on maize plants [106]. Both strains produced high IAA and ACC deaminase that acted as crop bio-fertilizers under drought-stress conditions. *Streptomyces pactum* Act12 facilitated the plant growth of drought-stress wheat seedlings, with significant increases in shoot fresh weight, shoot length, root length, and total soluble sugar content in wheat leaves, while decreasing malondialdehyde content [107]. *Streptomyces albidoflavus* OsiLf-2 produced abundant osmolytes, including proline, polysaccharides, and ectoine that significantly improved the osmotic-adjustment ability of the rice host by increasing proline and soluble sugar contents of rice under drought and salt stresses [108].

*Streptomyces* sp. RA04 and *Nocardiopsis* sp. RA07 enhanced cadmium accumulation, chlorophyll pigments, antioxidant enzymes, and growth of *Sorghum bicolor* under different abiotic stresses [109]. Siderophore-producing *Streptomyces phaeogriseichromatogenes* COS4 with a strong Cd tolerance potential significantly increased root-to-tip length and total dried weight of sunflower [110]. *Rhodococcus erythropolis* MTCC 7905 reduced substantial amounts of Cr(6^+^) to Cr(3^+^) at 10 °C and also increased the growth of *Pisum sativum* [111].

## 5. Genomics Approaches to Understand Actinobacteria-Mediated Alleviation of Abiotic Stress in Plants

The high-throughput sequencing approach makes it simple to obtain high-quality bacterial genome sequences [112], and genomics-based technologies have shown a significant impact on crop improvement initiatives, particularly in understanding the mechanism of microbe-mediated abiotic stress alleviation and adaptation in plants (Figure 2).

An endophytic PGP halotolerant *Streptomyces* sp. KLBMP 5084 alleviated salt stress of the halophyte *Limonium sinense* under greenhouse conditions [113]. Genome analysis of *Streptomyces* sp. KLBMP 5084 revealed the existence of genes for N_2_-fixation (*nifU*), IAA synthesis (*iaaM*), siderophores (*rhbCDEF*), phosphate solubilization, ACC deaminase, pyridoxal, and hydrogen cyanide. Additionally, genes for hydrolytic enzymes including chitinase, *β*-glucosidase, lipase, cellulose, protease, and amylase, were found. Potential biosynthetic gene clusters to produce secondary metabolites were discovered, including Type I, Type II, and Type III polyketide synthases (PKSs), non-ribosomal peptide synthetases (NRPSs), and hybrid NRPS-PKSs. There were also genes associated with hyperosmotic and oxidative stress, including superoxide dismutases, peroxidases, and catalases. *Streptomyces* sp. KLBMP 5084 was reported to encode proteins that aid in heavy metal resistance, as well as cold and heat shock proteins [113]. Similarly, genome analysis of a salt-tolerant deep-sea actinobacterium, *Dermacoccus abyssi* MT1.1^T^, was conducted to understand PGP and salt stress mitigation in tomato seedlings [97]. Genome study revealed the existence of genes involved in tryptophan biosynthesis as well as plant nutrient acquisition, including iron, phosphorus, and nitrogen. Genes related to ammonium assimilation, phosphate metabolizing enzymes (alkaline phosphatase, inorganic pyrophosphatase), poly-phosphorus hydrolyzing enzymes (polyphosphate kinase, polyphosphate glucokinase, and exopolyphosphatase), and the uptake and transport of inorganic phosphate as well as subsystem genes involved in the response to osmotic stress were noticed. Genes related to the glycerol uptake facilitator protein, ectoine biosynthesis, and oxidative stress were also detected [97].

Genome analysis of the plant-beneficial endophytic *Streptomyces chartreusis* WZS021 towards critical function in sugarcanes under drought stress was investigated [114]. The genome contained genes involved in plant growth promotion including nitrogen fixation, ACC deaminase, IAA secretion, Na^+^, Ca^2+^, and K^+^ transporters; important enzymes such as cellulase, chitinase, xylanase, glucoamylase, *α*-amylase, malto-oligosyltrehalose trehalohydrolase, and lipase; and genes involved in phosphate transmembrane transporters. Moreover, genes contributing to plant stress resistance such as oxidoreductase encoding SOD, glutamate dehydrogenase, succinate-semialdehyde dehydrogenase, proline dehydrogenase, and choline dehydrogenase were also detected [114]. Another endophytic Streptomyces sp. GKU 895 isolated from sugarcane was evidenced to promote the growth of sugarcane under individual and co-inoculation with endophytic diazotrophs [115]. There are several genes encoding for PGP-traits in its genome especially bacterial stress-responsive genes including ACC deaminase, proline dehydrogenase, superoxide dismutase, and trehalose synthase [115].

A salt-tolerant *Streptomyces paradoxus* D2-8 from *Phragmites comunis* rhizosphere soil was reported to enhance the soda saline–alkali stress tolerance of soybean. Genes related to PGP-traits including IAA biosynthesis, ACC deaminase, and ammonia assimilation were found in the genome of *S. paradoxus* D2-8. Genes related to stress tolerance including osmolytes such as ectoine and genes involved in the production and uptake of choline and glycine betaine were also detected [116]. A detailed list of genes that help in alleviating abiotic stress in plants is in Table 1.

## 6. Transcriptomics and Proteomics Approaches to Understand Actinobacterial Alleviation of Abiotic Stress in Plants

One of the key approaches used to analyze plant-microbe interactions is the use of mRNA sequencing analysis and microarray techniques to collect transcriptome-level information [77,117]. Many studies have been conducted in this regard to better understand the mechanisms by which actinobacterial alleviation of abiotic stress in plants occurs (Figure 2, Table 2). The transcriptional responses of wheat roots to salt stress inoculated with *Arthrobacter nitroguajacolicus* were studied to identify the key genes and pathways involved in the salt tolerance of wheat [98]. Upregulation of genes involved in cell, cell part, and the metabolic process was observed in salt stress wheat. One of the most enriched pathways in salt stress plants inoculated with *A. nitroguajacolicus* was the phenylpropanoid pathway, which is responsible for lignin biosynthesis of the cell wall, antioxidant activity, and interactions with biotic and abiotic environments [118]. Cytochrome P450s and hemethiolate enzymes involved in redox reactions and a variety of biosynthetic pathways were found to be upregulated in salt-stress wheat inoculated with *A. nitroguajacolicus*. Ascorbate and glutathione peroxidase, known to protect plant chloroplasts through enhancing reactive oxygen species scavenging capability, were also upregulated. Na^+^ influx transporter and the tonoplast Na^+^/H^+^ antiporter involved in Na^+^ homeostasis and vacuolar compartmentation in salt stress wheat were observed. Genes related to nicotinamine synthase, oligopeptide transporters, ATP-binding cassette transporters, sugar/inositol transporter, and ATPase were upregulated. Similarly, transcriptomic analysis of *Arabidopsis thaliana* inoculated with endophytic *Arthrobacter endophyticus* SYSU 333322 and *Nocardiopsis alba* SYSU 333140 indicated that both strains were involved in enhancing salt stress ability [117]. Genes related to potassium ion uptake, peptide-methionine (R)-S-oxide reductase, and the biosynthesis of secondary metabolites and phenylpropanoid were upregulated. Genes related to hydroxyproline-rich glycoprotein were upregulated in *A. thaliana* inoculated with *A. endophyticus* SYSU 333322 when compared to that of *N. alba* SYSU 333140. Genes related to carotenoid biosynthesis and nitrogen metabolism were observed in *A. thaliana* inoculated with *N. alba* SYSU 333140 but not with *A. endophyticus* SYSU 333322 [117]. This suggests that salt stress alleviation mechanisms by Actinobacteria were different from strain to strain.

A halotolerant *Streptomyces* sp. KLBMP5084 obtained from the root of halophyte *Limonium sinense* was evaluated to enhance the salt stress of tomato seedlings [119]. Transcriptome analysis revealed that genes related to secondary metabolites, such as isoquinoline alkaloid and betalain biosynthesis, were upregulated in tomato leaves treated with *Streptomyces* sp. KLBMP5084 under salt stress. Genes involved in chlorophyll a-b binding protein synthesis, auxin-responsive protein IAA29, and zeatin O-xylosyltransferase were upregulated, whereas cytokinin dehydrogenase and ethylene-responsive transcription factor were downregulated. The synthesis of auxin and cytokinin inhibited the synthesis of ethylene, promoted cell division, and accelerated cell growth, which contributed to the salt stress tolerance of tomato seedlings [119]. The effect of drought stress on the sorghum root microbiome was evaluated using meta-transcriptome analysis [121]. It was found that drought-induced shifts in rhizosphere function were driven by large changes in actinobacterial gene expression across nearly all gene ontology (GO) functional categories such as carbohydrates and amino acid transports and metabolisms, and by elevated expression of ABC transporters [121].

Understanding the effect of drought on plants necessitates assessing drought response in a variety of conditions; in this regard, identification of changes in *Populus deltoides* transcriptome and phytobiome were analyzed during both acute progressive drought and cyclic drought at various severities [122]. It was noticed that ROS and superoxide were positively associated with the abundances of six significantly different taxa, including *Streptomyces.* Genes associated with general water deprivation significantly abundant in cyclic drought were positively associated with seven taxa, *Rutstroemia*, *Brettanomyces*, *Conidiobolus*, *Puccinia, Trichinella*, *Streptomyces*, and *Mesorhizobium*. Photosynthesis-related transcripts significantly abundant in cyclic drought were affected by six taxa, *Trichinella*, *Puccinia*, *Streptomyces*, *Brettanomyces*, and *Conidiobolus* [122].

Studies suggest that plants generally enrich actinobacterial abundance under drought stress [79,83]. Recently, the mechanism of how plants regulate the enrichment of Actinobacteria during drought has been proposed. Using time-series root RNA-Seq data, it was demonstrated that drought stress affected iron homeostasis within the root, and the loss of a plant phytosiderophore iron transporter affected the microbial community composition, leading to a significant increase in the drought-enriched lineage, Actinobacteria [123].

Proteomes from branchlets of plants nodulated by nitrogen-fixing *Frankia* (NOD^+^) and non-nodulated plants priming with KNO_3_ (KNO_3_^+^) were analyzed to understand the molecular basis of *Casuarina glauca* response to salt stress [120]. Among the 357 quantified proteins, 43 were regulated by salt stress in KNO_3_^+^ plants and 25 in NOD^+^ plants, with 19 of them shared by both groups. By increasing salt concentrations, the number of differentially expressed proteins gradually increased in both KNO_3_^+^ and NOD^+^ plants. Differentially expressed proteins were multifunctional and involved in carbohydrate metabolism, cellular processes, and environmental information processing. Changes in protein levels in KNO_3_^+^ plants were minimal at 200 mM NaCl but increased at 400 mM NaCl and 600 mM NaCl [120]. This observation strongly reflects *C. glauca* ability to cope with salt stress. At the two first salt stress levels, NOD^+^ plants had a higher percentage of differentially expressed proteins than KNO_3_^+^ plants, while at 600 mM the percentage was lower. These variations are most likely caused by the fact that NOD^+^ plants experienced double the stress from 200 to 400 mM NaCl treatments; the symbiosis was then turned to residual levels and eliminates the nitrogen supply [120,124].

## 7. Conclusions and Future Perspectives

Plants are subjected to a variety of environmental stresses (water, salt, light, temperature, and nutrients), which reduce and restrict plant growth and productivity. Plants coexist with a wide range of microorganisms. Microbes that live alongside plants receive food and shelter from them, and in return secrete substances that help plant growth and overcome abiotic stress. The use of plant growth-promoting bacteria for enhancing plant health under abiotic stress has emerged as one of the most alluring methods for developing sustainable agricultural systems due to their eco-friendliness, low production costs, and decreased consumption of non-renewable resources. Actinobacteria are considered ideal candidates for plant growth promoters due to their profusion in soil and the rhizosphere, their capacity to invade plant roots and surfaces, and their ability to produce secondary metabolites. Abundant and diverse Actinobacteria are associated with plants and their action on plants has demonstrated plant growth directly, indirectly, or both. Plant-associated microbial diversity analysis (based on culture-dependent and 16S rRNA amplicon sequencing) under abiotic stress revealed a shift in actinobacterial abundance and diversity. Under abiotic stress, actinobacterial strains elevate plant growth and stress tolerance by altering gene expression involved in stress response. Genome analysis has revealed that under abiotic stress actinobacterial strains encode genes related to PGP activities. Various studies using transcriptomic and proteomic analysis confirm actinobacterial abiotic stress mitigation. Focusing on the reported culturable actinobacterial strains, only the tip of the iceberg has been explored for their PGP traits under abiotic stress and hence further studies need to be carried out for harnessing their PGP traits and abiotic stress mitigation.

## Figures and Tables

**Table 1:** Genomics approaches to understand actinobacterial-mediated genes alleviating abiotic stress in plants.

Stress	Actinobacteria	Genes	Host	References
Salinity	*Streptomyces* sp. KLBMP 5084	ACC deaminase, cold shock proteins, glycine betaine transport ATP binding protein, heat shock proteins, heavy metal resistance, hydrogen cyanide synthase, chitinase, *β*-glucosidase, lipase, cellulase, protease, and amylase, IAA biosynthesis, K^+^ transporter, Na^+^/H^+^ antiporters, oxidative stress response: SOD, POD, and CAT, phenazine biosynthesis, phosphate solubilization, pyridoxal biosynthesis lyase, succinate-semialdehyde dehydrogenase, trehalose synthase	*Limonium sinense*	[113]
	*Streptomyces* sp. GKU 895	ACC deaminase, ectoine biosynthesis, family 18 and 19 chitinases, IAA biosynthesis, mineral phosphate solubilization: isocitrate dehydrogenase, citrate synthase, and purple acid phosphatase, nitrogen metabolism, salicylate hydroxylase	Sugarcane variety KK3	[115]
	*Streptomyces paradoxus* D2-8	ACC deaminase, aldehyde dehydrogenase (NAD), ammonia assimilation, ectoine, IAA biosynthesis, choline and glycine betaine uptake	Soybean	[116]
	*Dermacoccus abyssi* MT1.1	Ammonium assimilation, betaine biosynthesis, catalase, choline and betaine uptake, ectoine biosynthesis, glutathionylspermidine synthase, glycerol uptake facilitator protein, IAA biosynthesis, iron acquisition and metabolism. nitrogen metabolism, osmotic stress response, oxidative stress response, phosphate metabolizing enzymes, phosphate solubilization, poly-phosphorus hydrolyzing enzymes, potassium homeostasis, proline synthesis, 4-hydroxyproline uptake and utilization, total soluble sugar production, trehalose biosynthesis, tryptophan synthesis, uptake and transport of inorganic phosphate	*Solanum lycopersicum*	[97]
Drought	*Streptomyces chartreusis* WZS021	ACC deaminase, choline dehydrogenase, glutamate dehydrogenase, cellulase, chitinase, xylanase, glucoamylase, *α*-amylase, malto-oligosyltrehalose trehalohydrolase, and lipase, IAA biosynthesis, ion transporter, Na^+^, Ca^2+^, and K^+^ transporters, oxidative stress response: SOD, phosphate transmembrane transporters, phosphate transport, proline dehydrogenase, succinate-semialdehyde dehydrogenase	Sugarcane varieties ROC22 and B8	[114]

**Table 2:** Plant–actinobacterial interaction confers salt stress tolerance in plants.

Plant	Actinobacteria	Pathways	Upregulation	Downregulation	References
*Triticum aestivum* L.	*Arthrobacter nitroguajacolicus*	Secondary metabolites, cysteine/methionine, diarylheptanoid, flavonoid/terpenoid/stillbenoid, glycerolipid, iron (Fe) acquisition, Na^+^ homeostasis, phenylpropanoid, photosynthesis, porphyrin/chlorophyll	Ascorbate/glutathione peroxidases, ATPase, cytochrome P450, hemethiolate enzyme, ion transporter, nicotinamide synthase, phosphatase, ABC transporter, sugar transporter, oligopeptide, amino acid/polyamine/folate-biopterin transporter	Cytochrome P450, metallothionein, RipA-like protein	[98]
*Arabidopsis thaliana*	*Arthrobacter endophyticus* SYSU 333322 *Nocardiopsis alba* SYSU 333140	Carotenoid, glycerolipid, secondary metabolites, phenylalanine, phenylpropanoid, nitrogen metabolism	Auxin binding, homeostasis, efflux, transport, chlorophyll a reductase, cytokinin dehydrogenase, DUF1399 domain-containing proteins, legume lectin family RING-finger E3 ligase, peptide-methionine (R)-S-oxide reductase, potassium ion uptake, hydroxyproline-rich glycoprotein	Phosphate starvation	[117]
*Solanum lycopersicum* cv. Jingpeng No.1	*Streptomyces* sp. KLBMP5084	Betalain synthesis, isoquinoline alkaloid, photosynthesis-antenna proteins, zeatin biosynthesis, protein processing in endoplasmic reticulum	Auxin-responsive IAA29, BOI-related E3, ubiquitin-protein ligase 3, calcineurin-like phosphoesterase, chitinase, chlorophyll a-b binding protein, 4,5-DOPA dioxygenase extradiol, elongation factor protein, glucan endo-1,3-beta-glucosidase, glutamine synthetase, linoleate 13S-lipoxygenase 2-1, peroxidase, glutathione S-transferase, receptor-like serine/threonine-protein kinase, salicylic acid carboxyl methyltransferase, zeatin O-xylosyl transferase	Cytokinin dehydrogenase, ethylene-responsive transcription factor	[119]
*Casuarina glauca*	*Frankia*	Amino acids, carbohydrates, metabolic pathways, secondary metabolites, cysteine/methionine, energy metabolism, lipid metabolism, seleno compound, protein processing in the endoplasmic reticulum, plant-pathogen interaction	ROS defence, monodehydro ascorbate reductase, temperature-induced lipocalin, thioredoxin-dependent peroxidase, Photosynthesis, quinone-oxireductase, thylakoid luminal 19 kDa, stress-responsive proteins, lipocalin, universal-stress protein, thaumatin		[120]

## Data Availability

Not applicable.
